# Lentiviral vector delivery of short hairpin RNA to NgR1 promotes nerve regeneration and locomotor recovery in injured rat spinal cord

**DOI:** 10.1038/s41598-018-23751-2

**Published:** 2018-04-03

**Authors:** Xiaoyang Zhao, Zhiming Peng, Lingli Long, Ningning Chen, Haichong Zheng, David Y. B. Deng, Yong Wan

**Affiliations:** 1 Department of Spine Surgery, The First Affiliated Hospital, Sun Yat-sen University, Guangzhou, 510080 China; 2 Department of Translational Medicine Center Research Laboratory, The First Affiliated Hospital, Sun Yat-sen University, Guangzhou, 510080 China; 3 Scientific Research Center and Department of Orthopedic, The Seventh Affiliated Hospital, Sun Yat-sen University, Shenzhen, 518107 China; 4 Department of Medical Intensive Care Unit, The First Affiliated Hospital, Sun Yat-sen University, Guangzhou, 510080 China

## Abstract

Nogo receptor 1 (NgR1) is a high-affinity receptor of myelin-associated inhibitors (MAIs), and suppresses neurogenesis. Lentiviral vector are commonly used to alter the expression of targeted genes. However, little is known about the potential function of lentiviral vector harboring NgR1 shRNA (LV-NgR1 shRNA) on neurogenesis in spinal cord injury (SCI). In this study, the rats were randomly divided into three groups: including the LN (LV-NgR1 shRNA injection), LC (LV-control shRNA injection) and Sham (laminectomy only). Eight weeks post-injection of LV, spinal cords were examined by histology for changes in cavity size and by immunohistochemistry for changes in expression of NgR1, cell apoptosis, astrocytes, neurons and myelination. Motor function was assessed using the Basso, Beattie and Bresnahan (BBB) locomotor scale. Animals that received LV-NgR1 shRNA remarkably improved the motor function. These animals also showed an increase in levels of nerve fibers, synapses and myelination, a decrease in levels of lesion cavity and cell apoptosis at 8 weeks post-treatment. These findings give evidence that NgR1 may be a promising target for SCI treatment.

## Introduction

Spinal cord injury (SCI) occurs worldwide with an estimated annual incidence of 15–40 cases per million, and is associated with severe physical, psychological, social, and economic burden on patients and their families^1^. SCI interrupts descending motor tracts and causes persistent functional deficits due to the absence of spontaneous axon regeneration and the formation of large cavities. Life-long time function impairment can ensue^2^.

Myelin-associated inhibitory components include Nogo-A, myelin-associated glycoprotein (MAG), and oligodendrocyte-myelin glycoprotein (OMgp) which can limitied axonal growth and neurological recovery after SCI^3–5^. Therefore, blocking the effects of these factors may cause a better outcome of functional recovery after SCI.

Nogo-66 receptor 1 (NgR1) attracts increasing attention as a converging point for modulating the effects of myelin-associated inhibitory ligands in the central nervous system, which can active RhoA, an intracellular molecule, to inhibit axonal re-growth and cell survival after traumatic injury^6–8^. In the rat model of SCI, using an NgR1 antagonist or NgR1 knockout leads to improved nerve function and more sprouting axons are found in animals after treatment^9–11^.

Conventional methods to delivery therapeutic agents for SCI, for example, administration and intravenous infusion have multiple limitations, including elevated risk of infection, low effective concentration, quick drug degradation and reduced tissue penetration^11^. Using lentiviral vectors as an efficient delivery system that offers stable and long-term expression in postmitotic cells further enhances the applicability of RNA-based gene therapy for the CNS. Short-hairpin RNA (shRNA) carried by lentiviral vectors could be locally injected at the lesion site to specifically silence target genes for months efficiently, meeting the therapeutic requirements for neural dysfunctions since neural repair is slow and overcomes the problems mentioned before^9,12,13^.

Therefore, we hypothesized that local injection of lentiviral vetors encoding NgR1-shRNA may promotes nerve regeneration and functional recovery after spinal cord injury. To test this hypothesis, lentiviral vectors carrying NgR1-short-hairpin RNA was injected into the injury site in rat model of SCI to knockdown NgR1 expression and the effects of this treatment on neuro-regeneration were determined *in vivo*. This study provides a novel insights into establishing NgR1 as a therapeutic target for SCI.

## Result

### Functional Recovery

**Figure 1:**
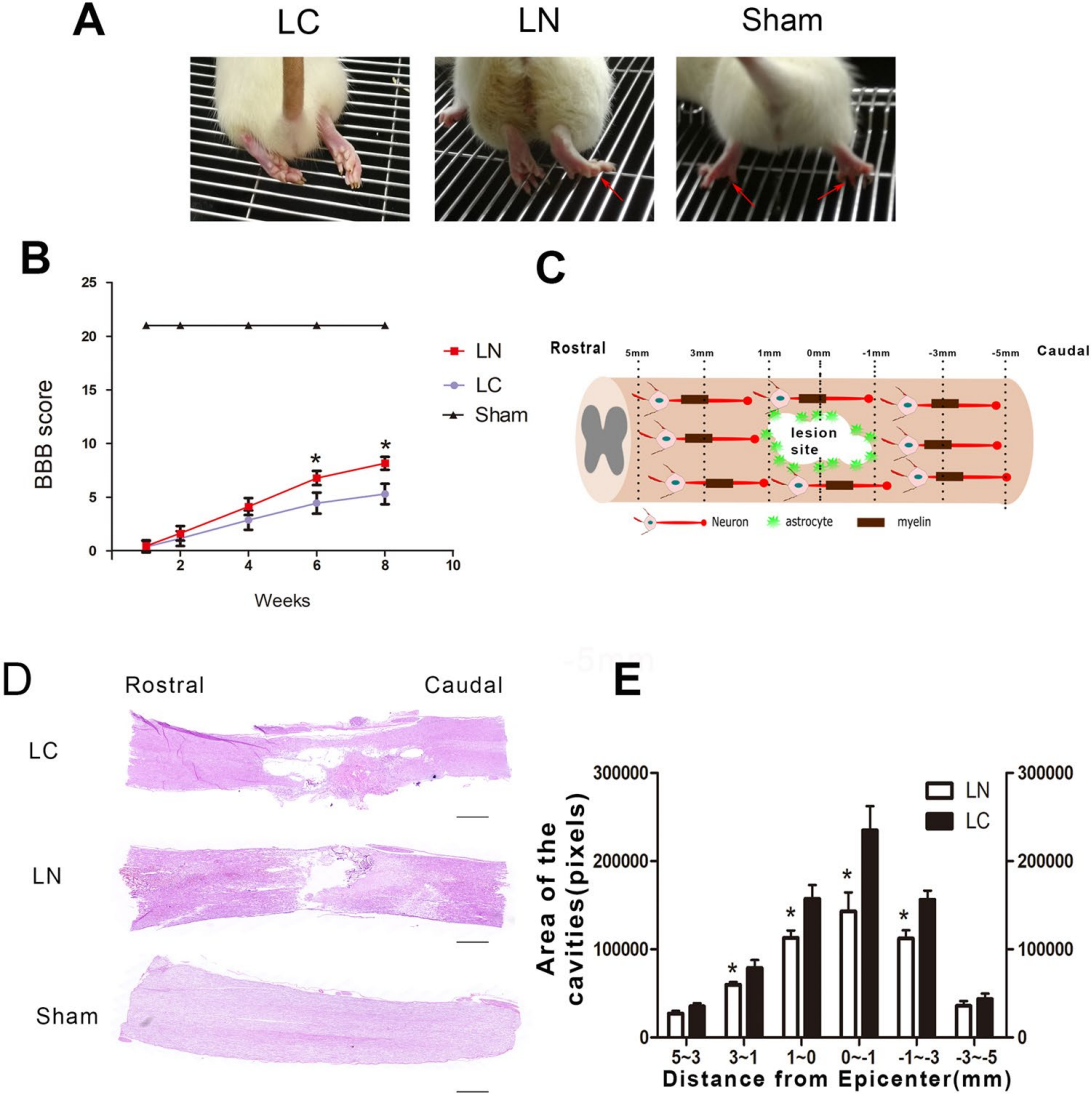
Motor function and tissue repair status in different groups. (**A**) Images showing hind limbs movements for the LC, LN, Sham groups. Red arrows indicate weight-supported stepping. (**B**) BBB scores in different groups. (**C**) Different distances from the lesion site diagram. (**D**) Longitudinal section of the sagittal plane after H&E staining, showing tissue continuity across the injury site with different cavity areas in each group. (**E**) Comparison of the cavity area between the LC and LN groups. Scale bar = 1000 μm, *P < 0.05.

In order to assess whether injection of LV-NgR1 shRNA enhances functional recovery after SCI *in vivo*. Weight-supported stepping, which which directly reflects motor function recovery, was observed at 8 weeks after injury. As shown in Fig. 1A, the sham group could easily use bilateral hind limbs to support their body weight, however, rats in the LC group could barely use their hind limbs to support their body weight. As expected, rats in the LN group could use one hind limbs or occasionally bilateral hind limbs to support their body weight. The results also showed that rats administered LV-NgR1-shRNA had significantly elevated BBB scores, from 6 to 8 weeks after SCI, compared with the rats treated with LV-control shRNA (P < 0.05) (Fig. 1B).

### Tissue Repair

We used HE stain to investigate the degree of tissue repairing. Eight weeks after SCI, both rostral and caudal stumps of the spinal cord were bridged in the LC and LN groups, suggesting that the animals could undergo tissue repair. Next, we measured the areas of lesion cavities at different distances (1 mm,3 mm,5 mm) rostral and caudal to the lesion site (Fig. 1C). In the sagittal plane, rats in the LN group had significantly smaller areas of cavities within 3 mm rostral and caudal to the lesion site compared the LC group (P < 0.05) (Fig. 1D,E).

### Apoptosis and NgR1 expression

**Figure 2:**
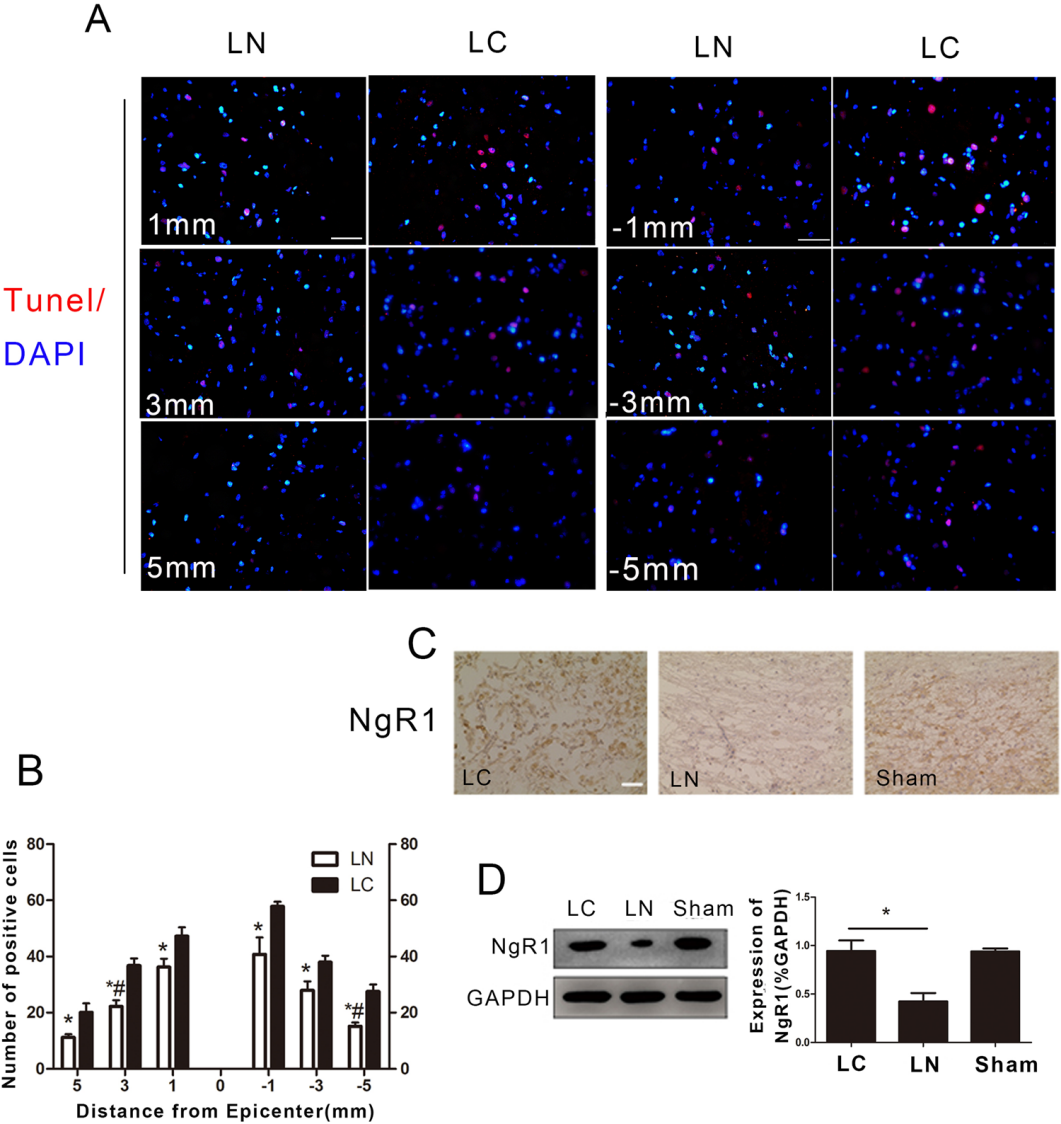
NgR1 expression and cell apoptosis in different groups. (**A**) TUNEL at different distances from the lesion site. (**B**) Quantification of TUNEL positive cells. (**C**) Immunohistochemistry was used to detect NgR1 expression at the lesion site. (**D**) Western blot was used to measure NgR1 protein levels in spinal cord samples in each group. Scale bar = 50 μm, *P < 0.05, *^,#^P < 0.01.

Pronounced apoptotic cell death detected by TUNEL was observed at different distances from the lesions site (Fig. 2A). The numbers of positive cells at different distances from the lesion site were significantly lower in the LN group than in the LC group (Fig. 2B). Next, we performed immunohistochemistry to further assess whether NgR1 expression at the lesion site was decreased 8 weeks after injection of LV-NgR1 shRNA. The results showed that NgR1 expression in the LN group was significantly reduced compared with LC and Sham groups (Fig. 2C). This finding was further supported by Western blot (P < 0.05) (Fig. 2D).

### Regeneration and Connection of Nerve Fibers

**Figure 3:**
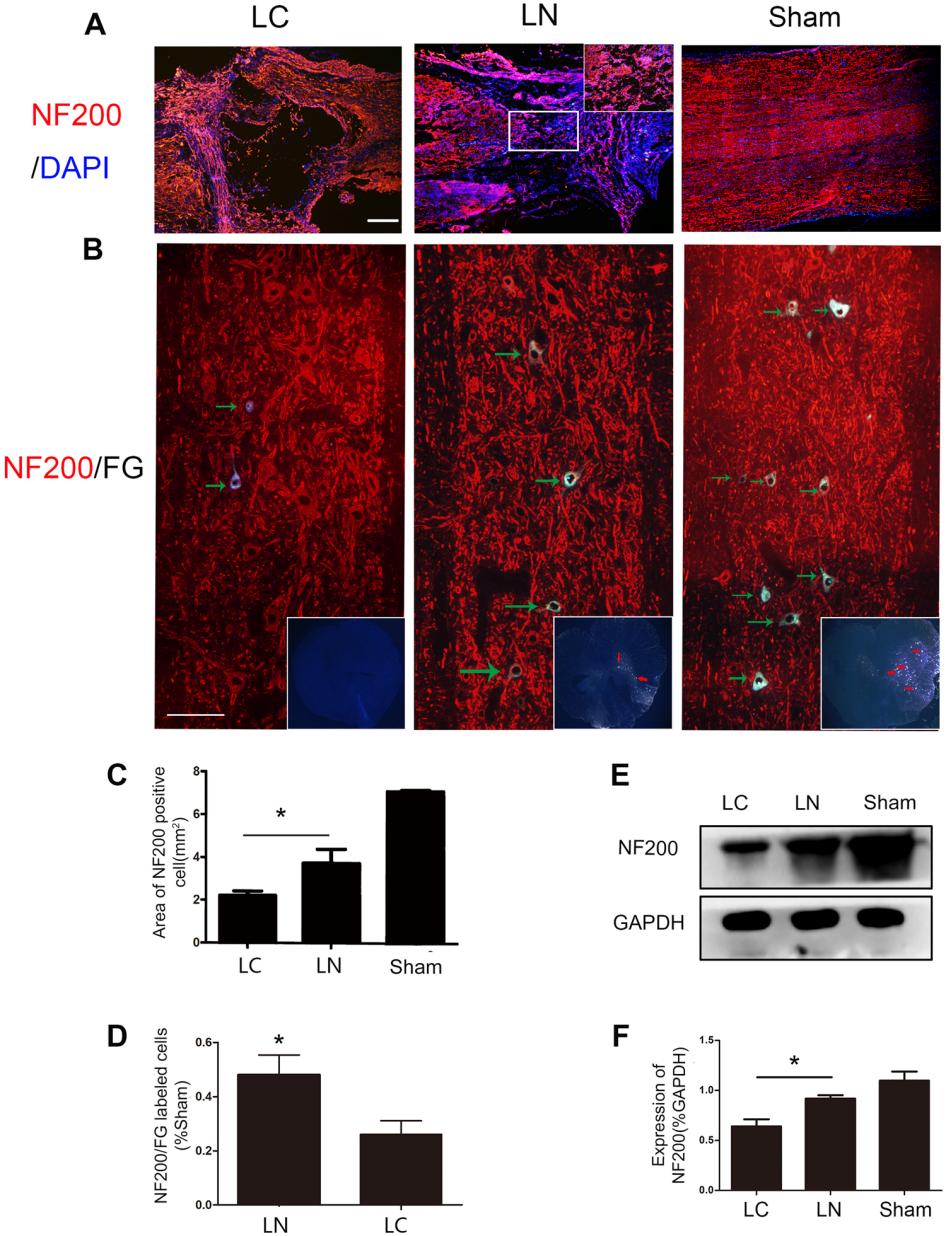
Regeneration of nerve fibers. (**A**) Immunofluorescent staining of the neural fiber maker NF200 in each group. (**B**) Immunofluorescent presenting, in the T7 segment spinal cord, FG and NF200 labeled neurons (indicated by green arrow) at the sagittal section and FG labeled cell (indicated by red arrow) at cross-section. (**C**) Quantification of the area of NF200 positive axons. (**D**) Quantification analysis of the number of NF200 and FG labled cells in LN group compared with LC group. (**E**) Western blot analysis of NF200 expression in the injured spinal cord. (**F**) Quantification of Western blot for NF200 expression. Scale bare = 100 μm, *P < 0.05.

Regeneration of nerve fibers labeled with NF200 was detected at the transection site in both groups. We found more positive fibers distributed within the injury site were found in the LN group compared with the LC group (P < 0.05) (Fig. 3A). The areas of NF200 positive fibers were significantly different between the LC and LN groups (Fig. 3C). These findings were also obtained by immunoblot (P < 0.05) (Fig. 3E,F). Furthermore, at T7 spinal cord, the FG and NF200 labeled neurons were more frequently observed in the LN group than LC group which means there are more nerve connections on both ends of the lesion site in the LN group (Fig. 3B,D).

### Neuron Regeneration

**Figure 4:**
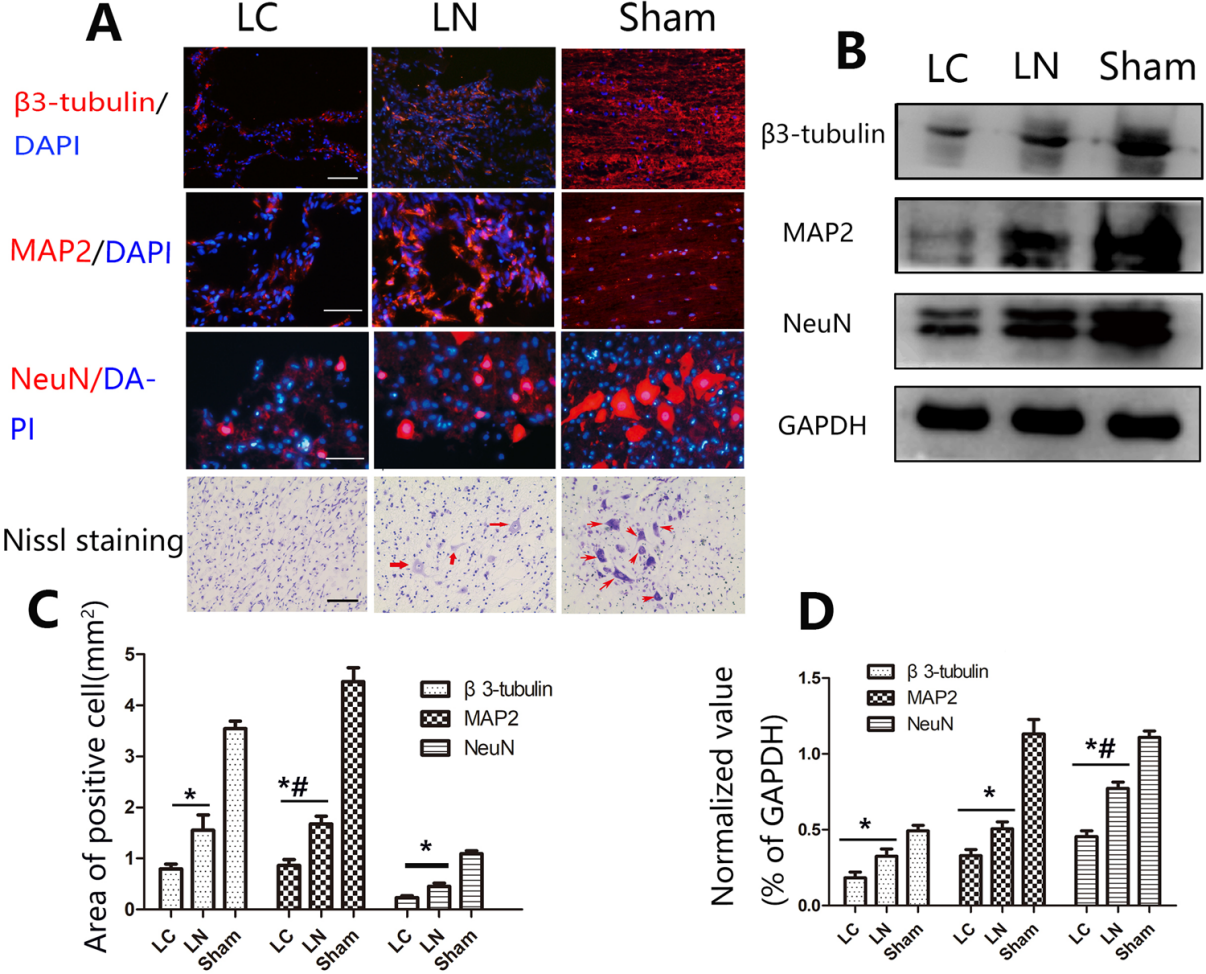
Regeneration of nerves in the injured area. (**A**) Immunofluorescent staining for the markers of newly formed and mature neurons (β3-tubulin and MAP2, respectively), and of functional neurons (NeuN), which were also identified by Nissl staining at the injury site. (**B**) Western blot analysis of NeuN, β3-tubulin, and MAP2 protein expression levels. (**C**) Quantification of the areas of NeuN, β3-tubulin, and MAP2 positive cells. (**D**) Quantification of Western blot for NeuN, β3-tubulin, and MAP2 expression. Scale bare = 50 μm, *P < 0.05, ^*,#^P < 0.01.

**Figure 5:**
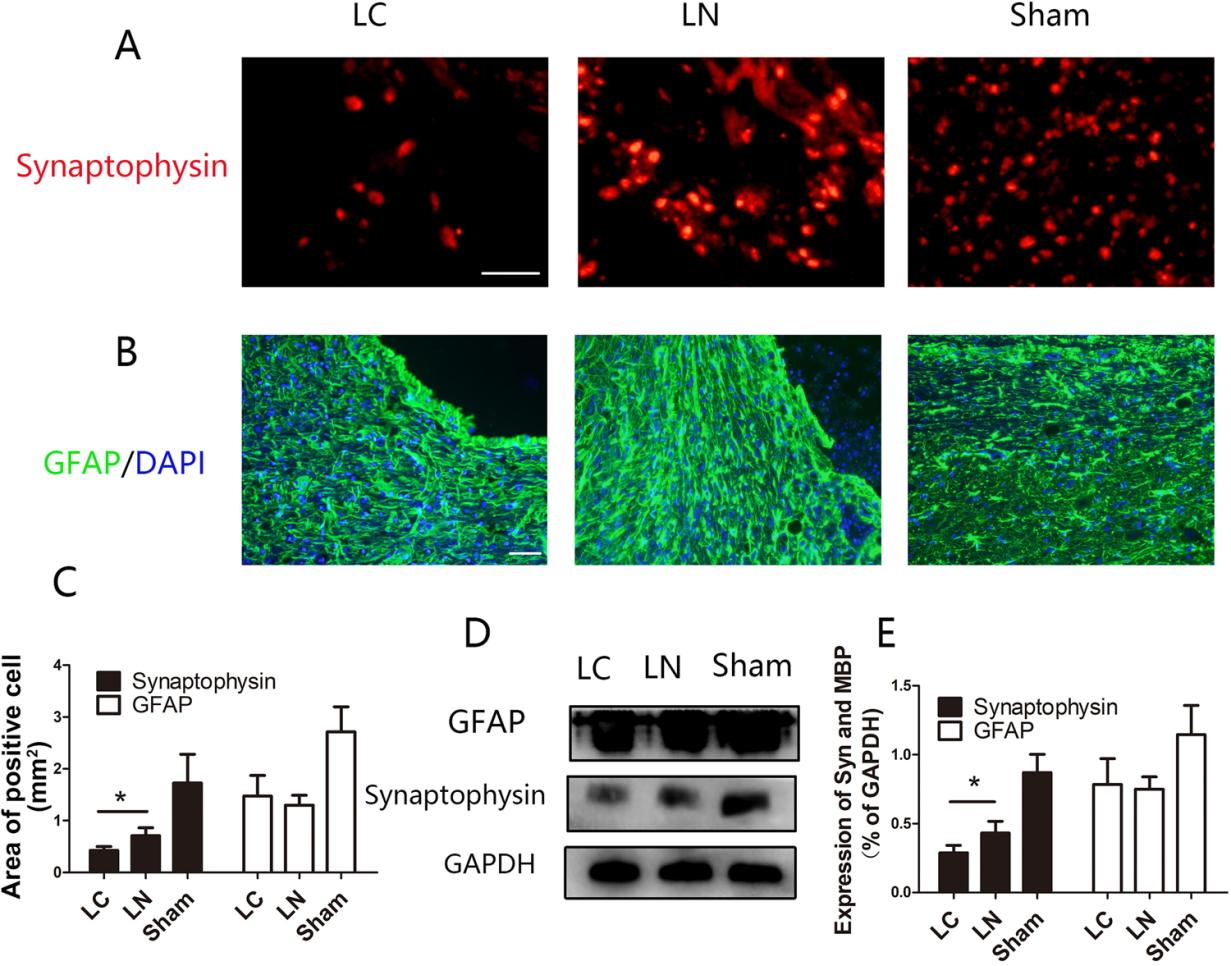
Assessment of synaptic regeneration and astrocyte responses. (**A**) The synapse marker Synaptophysin was detected in injured areas of all samples. (**B**) Micrographs showing GFAP labeled astrocytes adjacent to the central cavity. (**C**) Quantification of Synaptophysin positive areas and GFAP positive cells. (**D**) Western blot analysis of Synaptophysin and GFAP protein expression levels. (**E**) Quantification of D. Scale bare = 50 μm, *P < 0.05.

Neuronal cell death and suppressed nerve regeneration at the injury site occurring after SCI have been confirmed by many studies, and contribute to low function recovery^14,15^. We examined whether LV-NgR1-shRNA treatment could increase the number of neurons and improve nerve regeneration at the lesion site. Next, NeuN (marker for functional neurons) and β3-tubulin/MAP2 (newborn/mature neuronal markers) were assessed. The results showed more NeuN positive cells at the injury site in the LN group compared with the LC group (P < 0.05) (Fig. 4A,C). This finding was also confirmed by Western blot and Nissl staining. (P < 0.05) (Fig. 4A,B,D). Quantification of regeneration area and the markers of mature neurons (β3-tubulin and MAP2) demonstrated that rats in LN group had significantly more nerve regeneration compared with the LC group (P < 0.05) (Fig. 5A). This finding was also confirmed by Western blot (Fig. 4B–D).

### Astrocytic Response and Synapse Formation

**Figure 6:**
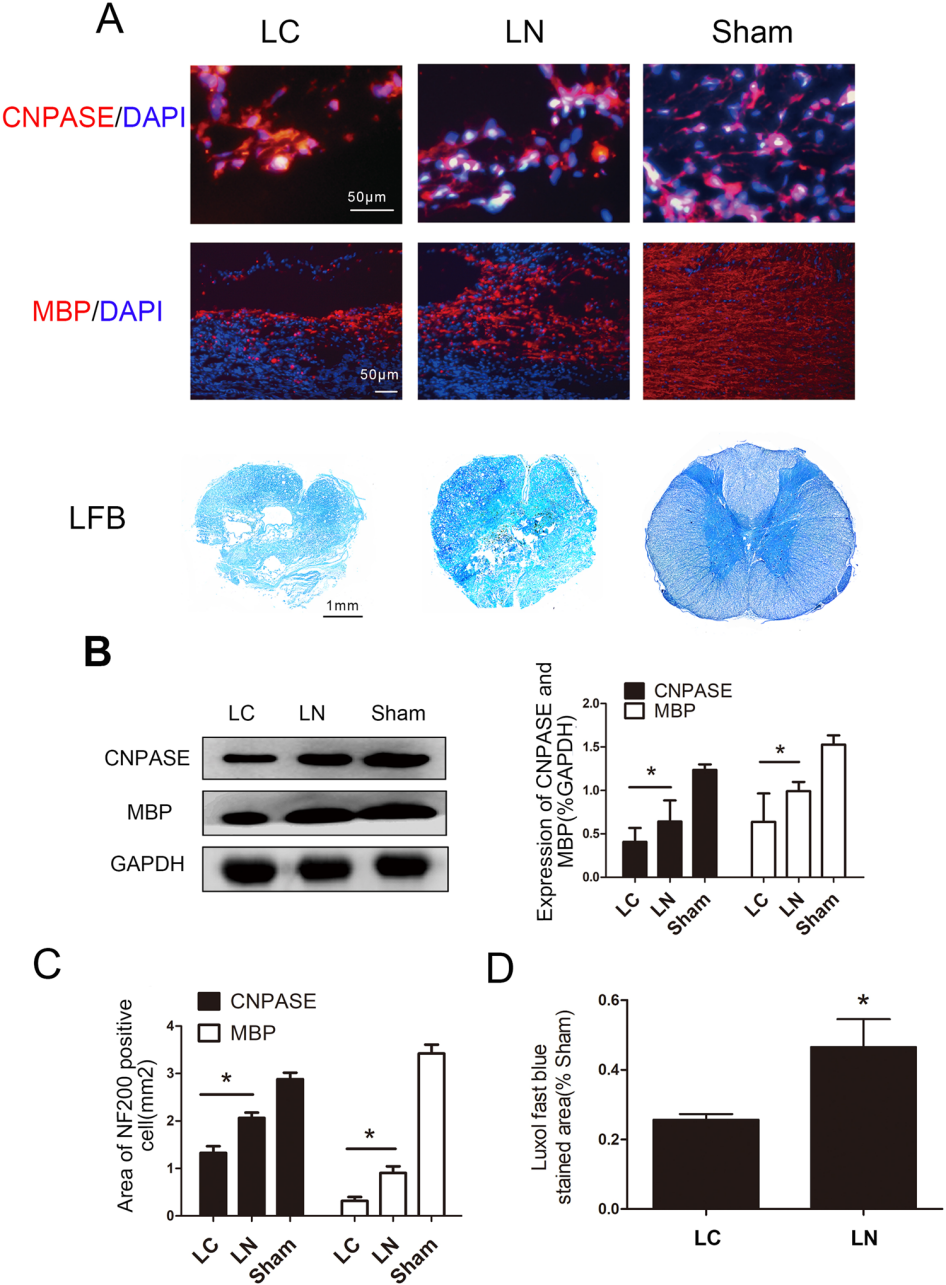
Detection of oligodendrocytes and myelin regeneration. (**A**) Oligodendrocytes were detected by the marker CNPase, and myelinated fibers by MBP immunostaining and LFB staining. (**B**) Western blotting analysis and quantification of CNpase and MBP expression. (**C**) Quantification of areas of CNPase and MBP positive cells. (**E**) Quantification of LFB stained areas, *P < 0.05.

Synaptophysin was used as a marker of synapse to detect synaptic connections between newly formed neurons. Synaptophysin expression was observed within the transection site in both groups (Fig. 5A). Synaptophysin levels at the injury site were more elevated in the LN group compared with the LC group (Fig. 5C). These findings were confirmed by immunoblot (P < 0.05) (Fig. 5D,E). Uncontrolled form of reactive astrogliosis that typically occurs around the injury site which can form glial scar to inhibit axon growth after SCI^16^. GFAP is a maker of astrocytes. GFAP positive cells were evaluated around the lesion site 8 weeks after surgery (Fig. 6B). Quantification of the GFAP positive area showed no significantly significant difference between the LC and LN groups (P > 0.05) (Fig. 5C). These findings were confirmed by immunoblot (P < 0.05) (Fig. 5D,E).

### Oligodendrocytes Survival and Remyelination

It was demonstrated that inflammation and other factors can cause oligodendrocytes apoptosis after SCI^17^. Whether LN increased the survival of oligodendrocytes at the injury site was assessed, using the oligodendrocyte marker. CNPase. The results showed more CNPase positive cells in the LN group compared with the LC group (Fig. 6A). This finding was also confirmed by immunoblot (Fig. 6B). Myelin is essential for nerve signal conductivity. Meanwhile, demyelination usually accompanies SCI. Therefore, The results showed only a few myelinated fibers in the LC group, while MBP expressing fibers were overtly present in the LN group (P < 0.05) (Fig. 6A) and quantitation demonstrated a significant difference between the two groups (Fig. 6C). At cross section, we used LFB to detect remyelination in each group. The result showd that more LFB stained area was observed in LN group than LC group (P < 0.05) (Fig. 6A,D). Finally, Western blots yielded a similar result (Fig. 6B).

## Discussion

In this study, we found that local injection of lentiviral vectors encoding NgR1-shRNA decreased NgR1 expression and promotes nerve regeneration and functional recovery after SCI in rats. Our findings provided the evidence that promoting nerve regeneration by inhibiting NgR1 expression worth to be a consideration for SCI in the future.

Compared with routine drug administration, genes delivered by lentiviral vectors in models of neuronal disorders have many advantages such as high transfection rate as well as long term and stable gene modifications^18,19^. Neuronal repair requires a long period, and such advantages make lentiviral vectors an advanced tool to effectively repair SCI in the long term.

Fournie *et al*. first discovered the Nogo-66 receptor (NgR), which specifically binds to the Nogo-66 domain^20^. Then, several homologous NgRs have been consecutively discovered; the one binding to Nogo-66, MAG, and OMgp was coined NgR1, and restricts axonal and synaptic plasticity^21^.

This inhibitory effect was reversed by NgR1 knockout or using an NgR1 antagonist^10,22^. For example, in the SCI model, treatment with NgR1-Fc may lead to a significant recovery in locomotor function and increased the growth of axons^11^.

In this study, endogenous NgR1 expression was blocked in SCI rat models by injection of LV-NgR1 shRNA. As a result, mobility of the SCI rats was improved significantly. Moreover, more axonal sprouting and new born nerves were found in the injury areas. These findings suggest LV-NgR1 shRNA is a potential therapeutic tool for SCI, but the mechanisms still require to be further investigation.

LV-NgR1 shRNA also promotes recovery after SCI is by decreasing of cell apoptosis. Indeed, programmed cell death following SCI contributes to tissue injury^23^, local cell survival represents an important factor for recovery after SCI. NgR1 complex has been described as mediator of apoptotic cell death by activating RhoA and phosphorylating Jun N-terminal kinase (JNK) in CNS^24^. Blockade of NgR1 function was shown to alleviate retinal ganglion cell apoptosis and generates a positive effect on oxygen-glucose deprivation condition^25,26^. However, it remains unclear whether injecting LV-NgR1 shRNA can decrease cell apoptosis after SCI. In this study, less apoptotic cells stained by TUNEL were observed in the LN group with more neurons detected, compared with the LC treatment group at the injury site, which likely contributes to plasticity of neural networks. This result is consistent with pervious studies that blockading of NgR1 function was shown to alleviate the effects on the synaptic dysfunction and neuron loss^27,28^.

Remyelination is critical for reconstructing axonal conduction and nerve function recovery^29^. Previous findings revealed that Nogo receptor blockade improves remyelination in the white matter stroke model^30^. In addition, NgR1 antagonists promote oligodendrocyte survival and myelin regeneration^31^. In this study, there were more remyelinated/myelinated nerve fibers and oligodendrocytes around the injured site in the LN treatment group compared with the LC group. The current findings indicated that injected LV-NgR1-shRNA induced the generation of myelin sheaths, which likely help restore function to the injured spinal cord.

According to our research, although the number of astrocytes surrounding the lesion site had no significant different between LN and LC groups, the functional recovery had significant different between two groups. This indicated the role of astrocytes in SCI is unclear. On the one hand, glial scarring caused by extreme, uncontrolled form of reactive astrogliosis are widely considered as inhibitors of axon regeneration after SCI^32,33^. Strategies of attenuating astrocyte activation and glial scar formation are considered advantageous for axonal regeneration^34,35^. On the other hand, some research have showed that reactive astrocytes protect tissue and reducing secondary tissue degeneration and improving functional outcome after SCI^36^. Furthermore how NgR1 regulates astrocytes also unclear. Findings have revealed that NgR1 antagonists can prevent the transformation of oligodendrocyte progenitors into astrocytes^30^. However, it was also reported that more reactive astrocytes occurr at the injury site in NgR1 knockout mice after SCI^6^. These results indicated a potential but undefined function for NgR1 in CNS ailments via mediation of astrocyte formation, and further investigation to explore the exact mechanisms is necessary.

Taken together, the present study suggested that the therapeutic effects of injected LV-NgR1 shRNA could be attributed to increased axonal sprouting, enhanced remyelination, and decreased cell apoptosis. This research may provide a further understanding of establishing NgR1 as a therapeutic target for SCI in the future.

## Materials and Methods

All procedures experimental animals were had approval from the Care and Use Committee of Sun Yat-sen University (ethics number: SYXX2012-0081) and performed in accordance with the Guide to the Care and Use of Experimental Animals provided by the National Research Council (1996, USA).

### Lentiviral vector-NgR1 shRNA Construction

LV-NgR1 shRNA (5′-CGGCAACCGAATCTCTTAC-3′) was designed and produced by GeneChem (Shanghai, China) and ligated into pGV112 (Hu6-MCS-CMV-puromycin) (Fig. S1) (GeneChem, Shanghai, China)., as well as LV-control shRNA (5′-TTCTCCGAACGTGTCACGT-3′).The Blast results of these oligonucleotides was shown in Figs S2, S3. Lentiviral vector particles were then produced according to a previous report^37^, with a final titer of 1 × 10^9^ Tu/mL.

### Surgical Procedures

Spinal cord surgery was performed on 80 adult female Sprague-Dawley rats (200–250 g). The rats were randomly divided into three groups, including the LV-NgR1 shRNA treatment (LN group), LV-control shRNA treatment (LC group), and Sham (spinal cord exposure only) groups. Briefly, animals were sedated with 10% chloral hydrate (3.5 mL/kg) for skin preparation, placement of the rat and sterilization. Then, 15 minutes prior to surgery, rats were anesthetized and analgesia by intraperitoneal injection of ketamine (50 mg/kg). Laminectomy was carried out at the level of the 10^th^ thoracic vertebra (T10). Afterwards, the spinal cord was cut twice using scissors (once rostral to T10 and once caudal to T10) for complete transection, removing a 2 mm block of the spinal cord. Following hemostasis, the rats were injected with 10 µl of LC or LN vector suspension at a depth of 1 mm rostral and caudal to the injured site using a microsyringe. Then, the muscle and the skin were sutured using 5–0 sutures, and 1 mL of saline containing 1 × 10^5^ units of penicillin was intraperitoneally injected daily for 1 week to protect against infection and dehydration. Postsurgical care of SCI rats included manual bladder expression twice daily until bladder function restoration. Surgery was performed in a blinded manner.

### Basso-Beattie-Bresnahan Test

The Basso, Beattie, Bresnahan (BBB) locomotor rating scale is considered a reliable tool for evaluating impairments of hindlimb locomotor skills after thoracic spinal cord injury^38^. The BBB score includes the assessment of joint state, gait, coordination, and torso stability ranging from 0 (no limb movement or weight support) to 21 (normal locomotion). Rats were placed in an open experimental field and allowed to move freely for 5 min, and the crawling ability was assessed by the BBB scale Hindlimb motor behavior was evaluated weekly for 8 weeks, with tests carried out the same time/day and grading performed by two investigators blinded to grouping.

### Perfusion and Cryosection

8 weeks after spinal cord surgery, rats were submitted to anesthesia and transcardial perfused of 0.9% normal saline, and 1 cm of spinal cord samples containing the injured area was extracted from the animals for Western blot analysis. The remaining animals were further perfused with 400 mL 4% paraformaldehyde (PFA) in 0.1 M phosphate buffer solution (PBS; pH 7.4). After collection, the T8-L1 segments of the spinal cord were post-fixed with 4% PFA overnight and transferred to 30% sucrose for cryoprotection for 2–3 days at 4 °C. Then, embedded tissues were sliced at 10 μm transversely or longitudinally, followed by mounting on poly-lysine coated glass slides, stored at −20 °C until use.

### Histopathological Analyses

To assess the cavity area and myelination, rats (N = 5 per group) were sacrificed for hematoxylin-eosin (H&E) and luxol fast blue (LFB) staining at 8 weeks after SCI. T8-T11 longitudinal spinal cord sections from each group were stained with H&E and LFB following standard protocols and observed under a bright field. The cavity and LFB positive area of those images assessed using the NIH ImageJ software (Wayne Rasband, National Institutes of Health, Bethesda,MD). For neuron examination, transverse sections of the tissue surrounding the injured site were submitted to Nissl staining (Beyotime, Shanghai, China, C0117).

### Immunohistochemistry

The animals were sacrificed for immunohistochemical detection of NgR1 8 weeks after SCI. T8-T11 longitudinal spinal cord sections in each group were removed, heated in citric buffer (pH 6.0) once for 3 min in a microwave oven for antigen retrieval. The sections were then washed with distilled water, blocked with 3% hydrogen peroxide and incubated with rabbit anti-NgR1 (Abcam, Cambridge, UK, #ab26291) monoclonal antibody at a dilution of 1:100 at 4 °C for 12 h. This was followed by staining with EnVision-HRP secondary antibody (Dako, Carpinteria, CA, K4003) for 30 min at room temperature, washing with a 0.01 mol/L concentration of PBS, staining with 0.5% diaminobenzidine and counterstaining with Mayer’s hematoxylin. Then, the specimens were air dried and mounted with glycerol gelatin for observation by bright field microscopy.

### Immunofluorochemistry

Tissue sections from rats (N = 5 per group) were fixed in 4% PFA for 30 min and permeabilized with 0.3% Triton X-100 for 30 min. Blocking was performed with 5% normal goat serum for 1 h, and tissue sections were incubated by the following primary antibodies at 4 °C overnight: β3-tubulin (1:200, CST; #5568), glia fibrillary acidic protein (GFAP, 1:300, CST; #12389), 2′,3′-Cyclic nucleotide 3′-phosphohydrolase (CNPase; 1:100, CST; #5664), neuronal nuclei (NeuN, 1:200, CST; #24307), neurofilament 200 KD (NF200; 1:200, CST; #2836), microtubule-associated protein 2 (MAP2; 1:200, CST; #8707), Synaptophysin (1:200, CST; #5467), myelin basic protein (MBP, 1:200, CST; #2396), and NgR1 (Abcam, Cambridge, UK; #ab26291), respectively. After three washes with PBS, Alexa Fluor 594-conjugated goat anti-rabbit (1:500, Thermo Fisher, MA, USA) or Alexa Fluor 488 conjugated goat anti-mouse (1:500, Thermo Fisher) secondary antibodies were added for 1 h at room temperature. Prolong Gold antifade reagent containing 4′-6-diamidino-2-phenylindole (DAPI) (Thermo Fisher) was used for staining nuclear. Then, the total area of targeted marker-positive cells was determined in each visual field under fluorescence (Carl Zeiss Axio Observer Z1, Germany) with the ImageJ software. Five random fields in each section and five sections per group were examined independently by 2 observers blinded to grouping.

### TUNEL Detection of Apoptosis

Cell apoptosis was measured by terminal deoxynucleotidyl transferase-mediated deoxyribonucleotide triphosphate nick end-labeling (TUNEL, Roche, Basel, Swiss). Tissue sections from rats (N = 5 per group) were fixed with 4% paraformaldehyde for 10 minutes at room temperature and washed with PBS twice, followed by 10-minute incubation with 0.2% Triton X-100. After 3 washes, incubation with TUNEL reagent was carried out in the dark at 37 °C in a humidified atmosphere for 1 hour. Coverslips were washed with PBS, mounted on slides with DAPI, and analyzed by fluorescence microscopy. Finally, targeted positive cells in each visual field were evaluated in a blinded manner.

### Immunoblot

To extract total protein from the animals (N = 5 per group) at 8 weeks after SCI, 1 cm samples of spinal cord tissue encompassing the lesion underwent perfusion with normal saline and homogenization with a mortar, after which they were lysed in RIPA buffer (Santa Cruz Biotechnology) containing protease inhibitors (Complete; Roche). Protein concentration was analyzed by the bicinchoninic acid assay (Beyotime Biotechnology, Shanghai, China). Equal amounts of protein (20 µg) were resolved by 10% SDS-PAGE followed by transfer onto PVDF membranes (Millipore, Bedford, USA). Blocking with 5% BSA (Sigma, Darmstadt, Germany) for 1 h was followed by incubation with primary antibodies at 4 °C overnight. The primary antibodies (1:1,000) were raised against NgR1 (Abcam, UK), β3-tubulin (CST, USA), CNPase (CST, USA), GFAP (CST, USA), NeuN (CST, USA), NF200 (CST, USA), MAP2 (CST, USA), MBP (CST, USA), Synaptophysin (CST, USA) and GAPDH (CST, USA). After washing with TBST (Tris-HCl-based buffer with 0.2% Tween 20, pH 7.5), the membranes were incubated with peroxidase-conjugated secondary antibody for 1 h at room temperature and washed three times in TBST. Detection was performed with the enhanced chemiluminescence (ECL) system (Millipore, Bedford, MA, USA).

### Axonal Tract Tracing

Animals (N = 5 per group) were used for retrograde tracing at 7 weeks after SCI. Dorsal laminectomy was performed at T12, and 0.5 μl of Fluorogold (FG; Biotium, Fremont, CA; #80014) was injected into the spinal cord using a Hamilton syringe. One week after injection, the animals were perfused and the T7 segment of the spinal cord was removed, cryopreserved, embedded in OCT compound, and sliced into 10 µm frozen sections. A fluorescence microscope (Olympus, Tokyo, Japan) was used to detect FG-labeled neurons.

### Statistical Analysis

All data was expressed as means ± SD. All statistical analyses were performed with GraphPad Prism 6 (GraphPad Software. Inc., USA). The data were first assessed for normality by the Shapiro-Wilk test, and showed normal distribution. Results were analyzed using one-way ANOVA followed by Bonferroni post hoc tests for multiple comparisons. Comparative studies between the two groups were performed using Student’s t-test. P < 0.05 indicated statistical significance.

## Electronic supplementary material

supplemental information
