# Supplementary material for: Lentiviral vector delivery of short hairpin RNA to NgR1 promotes nerve regeneration and locomotor recovery in injured rat spinal cord
Source: Sci Rep. 2018 Apr 3;8:5447. doi: 10.1038/s41598-018-23751-2 (PMC5882972; doi:10.1038/s41598-018-23751-2)
Supplement: Supplementary file 1 — supplemental information [file 41598_2018_23751_MOESM1_ESM.pdf]

Title: Lentiviral vector delivery of short hairpin RNA to NgR1 promotes nerve regeneration and locomotor recovery in injured rat spinal cord.

Author Names: Xiaoyang Zhao, Zhiming Peng, Lingli Long, Ningning Chen, Haichong Zheng, David YB Deng*, Yong Wan,*

SUPPLEMENTAL INFORMATION

1. Blast Results

(1) Blast of control RNAi

Rattus norvegicus vav guanine nucleotide exchange factor 3 (Vav3), transcript variant X1, mRNA
Sequence ID: XM_017590775.1Length: 5116Number of Matches: 1
Related Information
Gene-associated gene details
Range 1: 638 to 651GenBankGraphicsNext MatchPrevious Match
Alignment statistics for match #1

Score	Expect	Identities	Gaps	Strand
28.2 bits(14)	3.3	14/14(100%)	0/14(0%)	Plus/Minus

```
Query  4    TCCGAACGTGTCAC  17
            ||||||||||||||
Sbjct  651  TCCGAACGTGTCAC  638
```

(2) Blast of NgR1 RNAi

Rattus norvegicus NOGO-66 receptor (NgR) mRNA, complete cds
Sequence ID: AF462390.1Length: 1422Number of Matches: 1
Related Information
UniGene-clustered expressed sequence tags
Map Viewer-aligned genomic context
Range 1: 195 to 213GenBankGraphicsNext MatchPrevious Match
Alignment statistics for match #1

Score	Expect	Identities	Gaps	Strand
38.2 bits(19)	1.1	19/19(100%)	0/19(0%)	Plus/Plus

```
Query  1    CGGCAACCGAATCTCTTAC  19
            |||||||||||||||||||
Sbjct  195  CGGCAACCGAATCTCTTAC  213
```

2. Lentiviral vector structure and detection NgR1 and NF200 in the injured area

Fig.S1

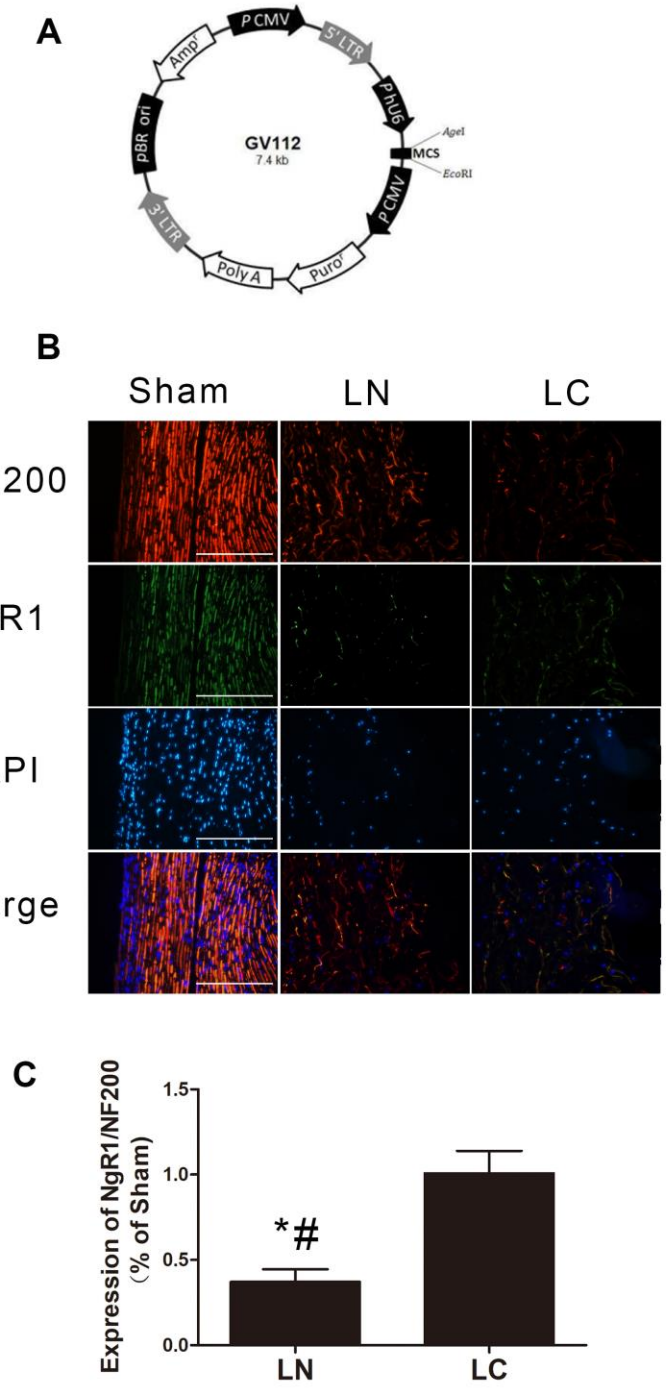

Figure S1: Lentiviral vector structure and detection NgR1 and NF200 in the injured area. A: Structure of pGV112.B：Immunofluorescent showing the NgR1 and neural fiber maker NF200 in lesion site at each group. C: Bars represented the ratios of

NgR1/NF200 expression. Note that NF200+ axons in the spinal cord normally express NgR1 and NgR1 expression was decreased in NF200 positive cell after treated LV-NgR1 shRNA. Bar=100μm. *#P<0.01
